# Aplicaciones del metaverso en medicina y atención sanitaria

**DOI:** 10.1515/almed-2024-0004

**Published:** 2024-02-19

**Authors:** Tim Hulsen

**Affiliations:** Data Science & AI Engineering, Philips, High Tech Campus 34, 5656AE Eindhoven, Países Bajos

**Keywords:** metaverso, telemedicina, gemelo digital, blockchain, medicina, sanidad

## Abstract

El metaverso es un mundo virtual, aún en proceso de desarrollo, que permite a las personas interactuar entre ellas, así como con objetos digitales de una forma más inmersiva. Esta innovadora herramienta aúna las tres principales tendencias tecnológicas: la telepresencia, el gemelo digital y la cadena de bloques. La telepresencia permite a las personas “reunirse” de manera virtual, aunque se encuentren en distintos lugares. El gemelo digital es el equivalente virtual y digital de un paciente, dispositivo médico o incluso de un hospital. Por último, la cadena de bloques puede ser utilizada por los pacientes para almacenar sus informes médicos personales de forma segura. En medicina, el metaverso podría tener distintas aplicaciones: (1) consultas médicas virtuales; (2) educación y formación médica; (3) educación del paciente; (4) investigación médica; (5) desarrollo de medicamentos; (6) terapia y apoyo; (7) medicina de laboratorio. El metaverso permitiría una atención sanitaria más personalizada, eficiente y accesible, mejorando así los resultados clínicos y reduciendo los costes de atención médica. No obstante, la implementación del metaverso en medicina y atención sanitaria requerirá una cuidadosa evaluación de los aspectos éticos y de privacidad, así como técnicos, sociales y jurídicos. En términos generales, el futuro del metaverso en el campo de la medicina parece prometedor, aunque es necesario desarrollar nuevas leyes que regulen específicamente el metaverso, con el fin de superar sus posibles inconvenientes.

## Introducción

**Figura 1:**
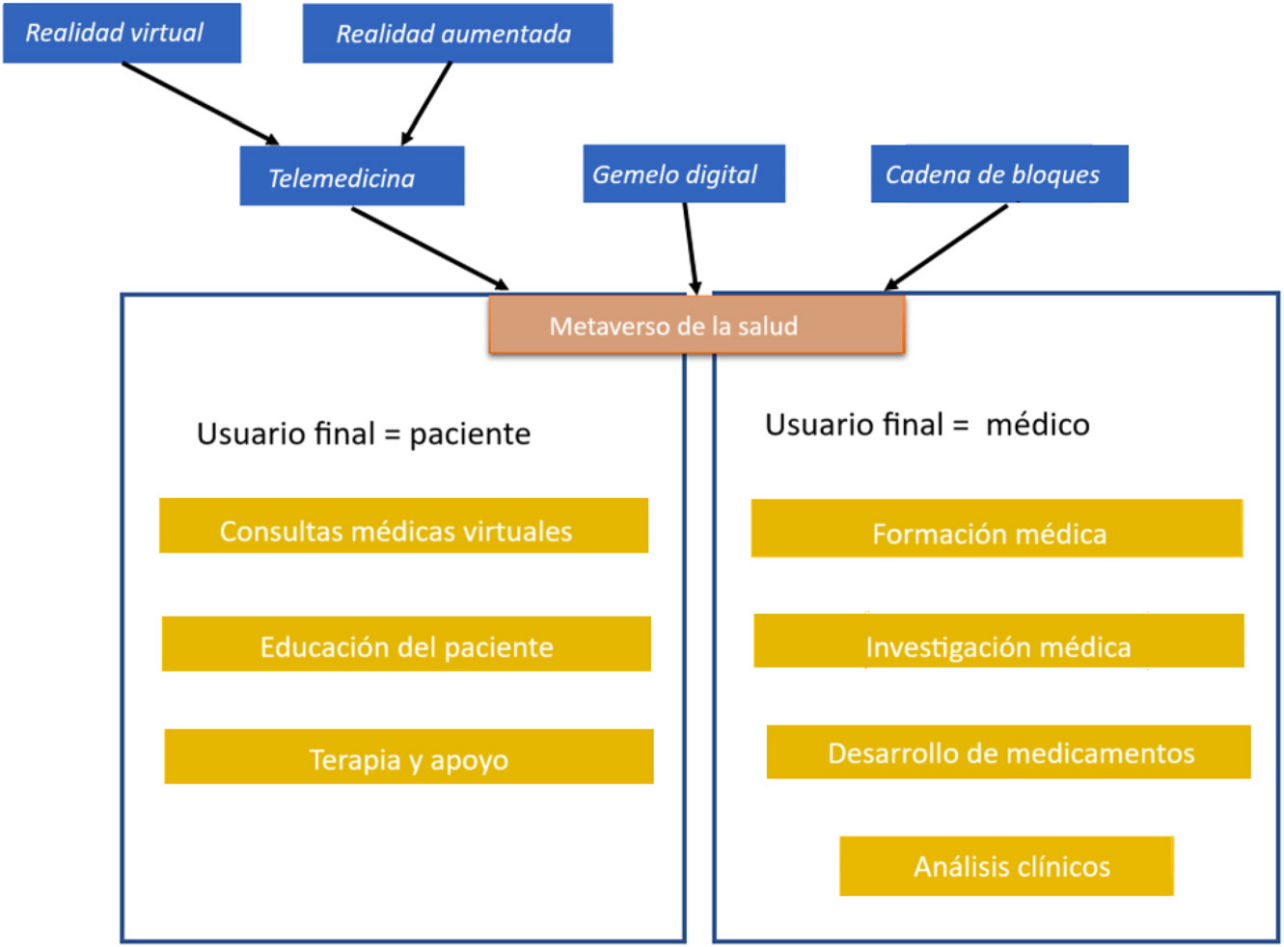
Componentes (azul) y áreas de aplicación (amarillo) del Metaverso Sanitario.

El metaverso es una tecnología emergente que podría revolucionar multitud de campos en el futuro. Se trata de un mundo virtual todavía en desarrollo, que permite a las personas interactuar entre ellas, así como con objetos digitales de manera más inmersiva que las actuales tecnologías basadas en el uso de pantallas e interfaces. Se puede considerar parte de la Web 3.0, que evoluciona hacia una web descentralizada en la que se emplean tecnologías como la cadena de bloques y la inteligencia artificial (IA) [1]. En el mundo de los videojuegos, el metaverso ya está presente [2], por ejemplo, en las plataformas de juegos en línea, como Second Life, Minecraft y Roblox, o en los videojuegos de rol multijugador masivo en línea (MMORPGs), como RuneScape o World of Warcraft. Otras áreas e industrias están siguiendo los pasos del mundo de los videojuegos introduciendo el metaverso, como el turismo [3] el marketing [4], el transporte [5] y la banca [6]. Incluso Facebook cambió en 2021 el nombre de su empresa matriz a “Meta”, creando un enorme efecto publicitario sobre el metaverso [7]. En la Figura 1 [8] se muestra un ecosistema de metaverso desarrollado por Faraboschi y col. [9], en el que se muestra una arquitectura informal, formada por componentes de economía, ecología, tecnología y sociedad. El metaverso también se está introduciendo en el ámbito de la medicina, aunque algunas de sus aplicaciones todavía están inmaduras.

En el metaverso convergen tres grandes tendencias tecnológicas [10], teniendo cada una de ellas el potencial de impactar en la medicina de manera individual, pero juntas podrían crear nuevos canales de atención sanitaria, que podrían reducir costes y mejorar enormemente los resultados clínicos (Figura 1). Estas tendencias tecnológicas son la telepresencia/telemedicina, el gemelo digital (GD) y la cadena de bloques. La telepresencia permite a las personas “reunirse” de manera virtual, aunque se encuentren en distintos lugares. Esto se puede lograr a través de la realidad virtual (RV, en la que se consigue una inmersión total del usuario), la realidad aumentada (RA, en la que el usuario ve una imagen real combinada con una imagen artificial) u otros medios. Además de la RV y la RA, Kye y col. [11] distinguen otros dos tipos: el registro digital de la vida personal, conocido en inglés como *lifelogging* (grabar, almacenar y compartir las experiencias del día a día e información sobre objetos y personas) y el mundo espejo, conocido en inglés como *mirror world* (en el que se refleja el mundo real tal cual es, pero integrando y aportando información sobre el entorno). En el campo de la medicina, la telepresencia se usa principalmente en la telemedicina, a través de la cual se proporciona asistencia médica de forma remota. Desde la pandemia de COVID-19, el uso de la telemedicina ha crecido exponencialmente [12]. El GD es el equivalente virtual y digital de un producto físico [13] y está compuesto por tres partes principales: el producto físico, el producto virtual y las conexiones de datos e información que los vinculan. En el campo de la medicina, un GD puede ser la representación de un paciente, dispositivo médico o incluso de un hospital. La cadena de bloques es un tipo de tecnología de registro contable distribuido (DLT, *Distributed Ledger Technology*), conocida principalmente por ser el concepto subyacente de las criptomonedas. Esta tecnología lleva un registro de transacciones (sin tocar los datos originales) y otros datos complementarios envueltos en varias capas de protección de datos (p. e. cifrado de datos). La cadena de bloques tiene dos importantes funciones en el metaverso: sirve como repositorio para almacenar datos en el metaverso y, además, puede servir como sistema económico para conectar el metaverso con el mundo real [14], por ejemplo, comerciando *tokens* no fungibles (NFT). En el campo de la medicina, la cadena de bloques es de especial interés, ya que ofrece la posibilidad de almacenar los informes clínicos de los pacientes y otros datos sensibles de forma segura (algo así como un “monedero” seguro, al igual que con las criptomonedas). Debido a su diseño descentralizado, los datos no pertenecen a ninguna organización, sino que permanecen bajo el control del paciente. El uso de las cadenas de bloques en la medicina favorece la transparencia y la inmutabilidad [15]. En medicina y atención sanitaria, el metaverso podría tener varias aplicaciones cuyo usuario final será el paciente o el profesional médico (Figura 1): consultas médicas virtuales, educación y formación médica, educación del paciente, investigación médica, desarrollo de medicamentos, terapia y apoyo, y medicina de laboratorio.

Chengoden y col. [15] y Yang y col. [16] ofrecen listas aún más detalladas de las posibles aplicaciones de esta tecnología. El metaverso tiene el potencial de permitir una atención sanitaria más personalizada y eficiente, mejorando así los resultados clínicos, al mismo tiempo que se reducen los costes médicos. Sin embargo, tal como ocurre con todas las nuevas tecnologías, también existen algunos riesgos potenciales e inconvenientes que deberán ser resueltos. A continuación, se muestra una descripción de cada una de las áreas de aplicación del metaverso, así como de los posibles desafíos que presenta, y de las perspectivas futuras.

## Áreas de aplicación

### Consultas médicas virtuales

Las consultas virtuales, en las que el médico puede llamar al paciente usando tecnologías de videoconferenciacomo Skype, Teams o Facetime, llevan muchos años en el mercado. Sin embargo, al utilizar estas tecnologías, el paciente puede tener cierta sensación de distancia con el médico. Con la RV, esta distancia puede percibirse más pequeña, dado que se trata de una experiencia mucho más inmersiva, lo que permite conversaciones más personales. Los primeros prototipos de RV que permitían las consultas médicas virtuales aparecieron hace algunos años, siendo un ejemplo el programa *Virtual Health Consultation* [17]. Zhang y col. [18] emplearon un programa de realidad mixta (RM) para las consultas virtuales y otros fines, en el que los objetos físicos y virtuales coexisten e interactúan en tiempo real. Kim y col. [19] desarrollaron una plataforma de servicios de asesoramiento psicológico espacial de RV, en la que los participantes crean y participan con sus propios avatares, y los asesores responden en diferentes entornos virtuales. Programas como estos podrían formar parte de un metaverso más amplio, permitiendo a los pacientes recibir asesoramiento médico y orientación, sin tener que salir de casa. Las consultas virtuales ahorran tiempo a los médicos y pueden resultar muy útiles a los pacientes con movilidad reducida. Estas podrán integrarse en un GD, lo que permitiría al paciente y al médico ver una representación digital del paciente durante la consulta, mejorando la comprensión por parte del paciente a la hora de entender la patología.

### Formación médica

El metaverso podría emplearse para proporcionar formación virtual y experiencias de simulación a los profesionales sanitarios, permitiéndoles practicar y mejorar sus habilidades sin poner en riesgo a los pacientes. Ya en el siglo pasado, se propuso realizar simulaciones de intervenciones quirúrgicas empleando la RV [20]. Actualmente, existen varios sistemas que posibilitan la formación y simulación en RV. Un ejemplo es la plataforma *Precision VR* de la compañía Surgical Theater, que crea modelos de RV de 360° específicos de un paciente a partir de tomografías volumétricas (p. e. TAC, RMN), que se pueden manipular y visualizar desde cualquier ángulo, a través de una pantalla táctil o unos auriculares de RV con mandos [21]. La plataforma de RV MetaMedics [22] proporciona formación en RV dirigida a cualquier tipo de profesional sanitario, incluyendo personal de enfermería, cirujanos y otros especialistas médicos. Estos sistemas formativos de RV presentan la principal ventaja de que pueden devolver información personalizada de forma inmediata a la persona que está realizando la formación [23]. Sandrone [24] muestra que juegos que utilizan el metaverso y avatares para llevar a cabo escenarios médicos se han probado con éxito en muchos campos médicos y en la educación médica tanto preclínica como clínica, mejorando los resultados de aprendizaje y mejorando la participación y colaboración de los estudiantes de medicina en un entorno de toma de decisiones libre de riesgos. Este tipo de tecnologías de “aprendizaje inmersivo” está evolucionando rápidamente y puede transformar la formación médica en un futuro cercano [25]. Estudios recientes muestran que muchos estudiantes de medicina prefieren las instituciones ciberfísicas a las instituciones convencionales [26]. Además de los sistemas de formación y simulación, también existen trabajos de RV de referencia. Por ejemplo, la compañía Medical Augmented Intelligence (MAI) creó “BodyMap” [27], una representación médicamente precisa del cuerpo humano que se puede manipular en RV en 3D, útil para fines formativos.

### Educación del paciente

El metaverso se podría emplear para ofrecer a los pacientes recursos formativos virtuales sobre temas como los hábitos de vida saludables y la prevención de enfermedades [28]. Del mismo modo, podría mostrar a los pacientes cómo va a ser la cirugía a la que se van a someter o los análisis que se han realizado en el laboratorio. El propósito de los llamados “servicios de asesoramiento al paciente previo a la cirugía” es disipar los temores de los pacientes, proporcionando información relacionada con la anestesia, el procedimiento quirúrgico y las complicaciones postoperatorias, y ayudarles a familiarizarse con el entorno en el que se va a llevar a cabo la intervención [29]. Algunas de las ventajas del asesoramiento virtual sobre el asesoramiento verbal son: (1) disminución de las posibles barreras idiomáticas; (2) ahorro de tiempo a los médicos; y (3) evitar a las personas con movilidad reducida tener que salir de casa. La compañía HealthBlocks ha lanzado una *app* basada en la tecnología de cadena de bloques, para ayudar a los usuarios a mantenerse activos y sanos [30]. Por otro lado, Healthify ha desarrollado un metaverso de deportes y salud basado en esta misma tecnología [31]. Los GD se podrían emplear para ayudar a los pacientes a conocer mejor su enfermedad mostrándoles una representación digital del paciente con la enfermedad.

### Investigación médica

El metaverso podría servir para simular procedimientos y tratamientos médicos, permitiendo a los investigadores probar y mejorar nuevos tratamientos e intervenciones. Esto resulta de especial utilidad en las enfermedades psiquiátricas, como el trastorno por déficit de atención e hiperactividad (TDAH) [32], la enfermedad de Alzheimer (EA) [33] o las enfermedades que provocan un deterioro de la movilidad, como la enfermedad deParkinson (EP) [34]. Respecto a la EP, se han realizado intervenciones de RV en forma de juegos inmersivos, mediante programas de entrenamiento individualizado que animaban a los pacientes a trabajar su movilidad y su memoria autobiográfica. Se demostró que el entrenamiento de rehabilitación con RV mejora la marcha y el equilibrio en los pacientes con EP, cuando se utiliza en combinación con la rehabilitación tradicional. Del mismo modo, el metaverso podría acelerar el desarrollo de los ensayos clínicos, rompiendo las barreras físicas y geográficas entre el clínico y los pacientes incluidos en un ensayo [35]. Zhang y col. [36] indicaron que la implementación de la tecnología de cadena de bloques en los ensayos clínicos ofrece una plataforma segura y transparente para la gestión de datos. Dicha plataforma puede favorecer una conducta eficiente y efectiva de los ensayos clínicos, mejorando la integridad y seguridad de los datos médicos, mejorando la confianza y aliviando la carga regulatoria. La cadena de bloques también se puede emplear para mejorar la seguridad de las historias clínicas digitales (HCD) [37], permitiendo a los investigadores extraer información de las mismas con riesgos de seguridad mínimos. Los GD se pueden emplear para recrear a los pacientes y los dispositivos médicos, permitiendo a los investigadores realizar estudios sin interferir directamente en el paciente [38]. Wang y col. [39] llaman al metaverso empleado en el ámbito sanitario “MeTAI” (“*medical techonology and AI*”) y defienden que este puede facilitar el desarrollo, diseño de prototipos, evaluación, regulación, traslado y mejora de la práctica médica basada en la IA, especialmente en el campo del diagnóstico y las terapias guiadas por la imagen.

### Desarrollo de medicamentos

El metaverso tiene el potencial de acelerar el desarrollo de medicamentos, mejorar la seguridad y eficacia de los nuevos fármacos y reducir el tiempo y el coste que conllevan la comercialización de nuevos fármacos. El metaverso puede permitir la creación de entornos virtuales donde los desarrolladores de fármacos pueden simular los efectos de los fármacos sobre el cuerpo humano. Estas simulaciones pueden ayudar a los investigadores a identificar los posibles problemas de seguridad y eficacia de los nuevos fármacos antes de probarlos en ensayos clínicos en humanos, pudiendo así reducir el tiempo y los costes asociados al desarrollo de medicamentos. En el diseño de fármacos, la RV no solo sirve para visualizar e interactuar con los GD de las moléculas, sino también para interactuar con simulaciones dinámicas moleculares “sobre la marcha” (también llamadas “Interactive Molecular Dynamics in VR”, “dinámica molecular interactiva en RV”, IMD-VR) [40]. Algunos ejemplos de programas para el desarrollo de medicamentos basados en RV son UCSF ChimeraX [41] y YASARA [42], a través de los cuales se puede realizar el acoplamiento, la simulación virtual de interacciones moleculares. Narupa iMD [43] es un programa de IMD-VR que permite a los usuarios colaborar en un único espacio de realidad virtual e interactuar con simulaciones moleculares en tiempo real. Con estas nuevas técnicas, las compañías farmacéuticas podrán completar sus estudios clínicos en semanas en lugar de meses o años, y an un coste reducido [44]. Además de GD de moléculas, también se pueden crear GD de órganos (p. e. el hígado [45]) o modelos animales (p. e. ratones [46]) para mejorar el proceso de desarrollo de medicamentos. El diseño y la selección de participantes para ensayos clínicos, esenciales para probar la seguridad y eficacia de nuevos fármacos, pueden mejorarse utilizando el metaverso. También se pueden utilizar simulaciones virtuales para diseñar ensayos clínicos más efectivos, pudiendo emplear entornos virtuales para facilitar la participación y compromiso de los participantes de los estudios. Del mismo modo, el metaverso puede favorecer la colaboración y comunicación de datos entre investigadores, permitiéndoles aunar recursos y conocimientos, con el fin de lograr un desarrollo de medicamentos más eficiente. Los investigadores de todo el mundo pueden colaborar en entornos virtuales para compartir datos, comentar hallazgos científicos y colaborar en proyectos de desarrollo de medicamentos y de bioinformática [47].

### Terapia y apoyo

El metaverso también puede ayudar a los pacientes que padecen algunos tipos de fobias (p. e. agorafobia [48], acrofobia [49], miedo a volar [50]) o trastorno de estrés postraumático (TEPT), a través de la ‘terapia de exposición’ [51], mediante la cual se expone a los pacientes a las situaciones que les provocan ataques de ansiedad. Normalmente, esto se tendría que hacer “*in vivo*”, esto es, el paciente tendría que afrontar su miedo en un contexto real. Sin embargo, también se puede utilizar un entorno de RV en el que se simula la situación “peligrosa”. Esta terapia de exposición mediante RV (ERV) es más segura, cómoda, controlable y costo-efectiva que su homóloga “*in vivo*”*.* Los últimos desarrollos en este campo se centran en hacer que la experiencia en RV sea lo más realista posible, con el fin de crear una “ilusión de personificación” mediante integraciones multisensoriales, en las que los pacientes se sienten como si realmente estuvieran en su cuerpo virtual (o “avatar”) [52]. Este tipo de “realidad virtual inmersiva” (RVI) ha mostrado resultados muy prometedores en personas con discapacidad intelectual [53]. También, el metaverso podría facilitar la rehabilitación y fisioterapia [54], ya que los pacientes podrían realizar ejercicios en RV para mejorar su movilidad y coordinación, desde la comodidad de sus hogares. Esto podría ser de especial utilidad para las personas que residen en entornos rurales o remotos, o para aquellas con problemas de movilidad que les dificultan los desplazamientos hasta un centro sanitario. A través del metaverso, también se pueden celebrar sesiones virtuales de terapia de grupo e individuales para pacientes con patologías crónicas o trastornos mentales. Estos grupos de apoyo proporcionarían una sensación de pertenencia y conexión a personas que no tienen acceso an un apoyo presencial.

### Medicina de laboratorio

Algunas de las aplicaciones del metaverso descritas anteriormente, como la educación y la formación, también se pueden introducir en el contexto del laboratorio médico, ayudando a los estudiantes, científicos y técnicos a aprender a colaborar de manera efectiva en el laboratorio. Por otro lado, los tres componentes del metaverso tienen algunos usos concretos en el laboratorio [55]. Por ejemplo, se pueden crear GD de los laboratorios y sus equipos [56], lo que permite la fabricación inteligente, el modelado *in silico*, la preevaluación y simulación de los ensayos y el rendimiento de dispositivos. La telepresencia/RV permite realizar visitas virtuales dinámicas a los laboratorios clínicos en 3D, así como el modelado digital de espacios, entornos y flujos de trabajo en el laboratorio [57]. Otra aplicación es un sistema de gestión de laboratorio remoto basado en la cadena de bloques [58], que permite la transferencia segura de datos entre equipos de laboratorio, así como entre técnicos y estudiantes. En el campo de la investigación en bioinformática y genómica, existen programas como BioVR [59], que permiten la integración y visualización de datos biológicos, todo asistido por RV. La RV se puede utilizar para el modelado molecular (véase la sección “Desarrollo de medicamentos”), el análisis multi-ómico, el modelado mesoscópico de cuerpos rígidos o incluso la visualización en 3D de una célula virtual [60]. La cadena de bloques también se utiliza en el área de la bioinformática, por ejemplo, para la transferencia segura de datos de secuenciación de ADN [61].

## Posibles retos

Cabe señalar que la implementación del metaverso en medicina y atención sanitaria requiere una cuidadosa evaluación de los aspectos éticos y relacionados con la privacidad, así como técnicos, sociales y regulatorios. Entre las posibles cuestiones éticas que plantea, se encuentran los aspectos relacionados con la integridad, ya que se puede difundir información falsa, realizar fraudes y violar los derechos de propiedad intelectual [62]. Un entorno digital como el metaverso también puede ser susceptible a la publicidad de productos no saludables [63]. Al igual que ocurre en las redes sociales, el metaverso debe regirse por unas directrices morales, con el fin de prevenir comportamientos inadecuados. Con respecto a la privacidad, el pirateo o *hackeo* (que provoca la pérdida de información personal sensible) resultaría difícil, debido a las propiedades inherentes a la cadena de bloques. No obstante, los usuarios aún muestran preocupación por la privacidad en el metaverso [64]. Dado que las leyes de privacidad del mundo real no serían aplicables a entornos virtuales [14], sería necesario desarrollar leyes de privacidad para el metaverso, con la colaboración de todos los agentes implicados. El uso del metaverso puede resultar problemático, cuando las personas lo utilizan para sustituir el contacto real físico humano, lo que provocaría sentimientos de soledad. Además, cuando las personas con trastornos mentales empiezan a utilizar el metaverso, pueden llegar a confundir el metaverso con la realidad, lo que desencadenaría otras patologías, como el ciber-síndrome o síndrome cibernético [65]. Entre las principales dificultades técnicas que plantea el metaverso, se incluyen la dificultad para garantizar la exactitud y fiabilidad de las simulaciones virtuales. Especialmente cuando se le da una aplicación médica, la RV precisa ser fiable, lo que aún no se puede garantizar actualmente [66]. Los equipos de RV/RA aún son costosos y pueden provocar efectos secundarios, como problemas oculares, tensión, fatiga y visión borrosa [24]. Finalmente, un gran número de las dificultades anteriormente descritas tienen implicaciones jurídicas. Las actuales leyes sobre tecnologías digitales resultan insuficientes a la hora de abordar problemas concretos relacionados con el metaverso. Por ejemplo, ¿quién tiene la responsabilidad en un mundo virtual? ¿Qué ocurre si un avatar (o un paciente virtual) comete un delito? Dado que los avatares están vinculados an una sola personal “real”, esta podría ser susceptible de acusación, pero ¿y si los avatares están parcialmente dirigidos por la IA [67]? Quizá se le debería dar personalidad legal aparte a los avatares [68], para diferenciarlos jurídicamente de los humanos. Al igual que se están desarrollando leyes para regular aspectos concretos de la IA [69], es necesario desarrollar leyes que regulen el metaverso, con el fin de crear un marco legal que regule los aspectos éticos, de privacidad, sociales y técnicos.

## Conclusiones y perspectivas

Mientras que el GD es una representación virtual de un solo paciente o dispositivo médico, el metaverso ofrece un entorno virtual más amplio con multitud de posibilidades. Las actuales aplicaciones del metaverso en el campo de la medicina se limitan a la formación, terapias y apoyo virtual, así como al desarrollo de medicamentos. Sin embargo, el futuro podría ser un hospital virtual, un laboratorio virtual o incluso un “continuo asistencial” virtual, esto es, un sistema integrado de atención, mediante el cual se guía y rastrea al paciente a lo largo del tiempo, a través de una gama integral de servicios sanitarios, que abarcan todos los niveles de intensidad asistencial [70], tanto en el hogar como en la clínica. El metaverso es muy adecuado para su uso en atención médica, ya que influye en varios mecanismos cognitivos clave: la experiencia de estar en un lugar (p. e., un hospital o laboratorio), la experiencia de estar en un cuerpo (p. e., el cuerpo de un paciente virtual), sintonía individual cerebro a cerebro (p. e., interacción médico-paciente), sincronía grupal cerebro a cerebro (p. e., el aula virtual) y emociones (p. e., regulación emocional durante las sesiones terapéuticas) (Tabla 1 de [71]). También implica algunos desafíos de tipo ético y relacionados con la privacidad, así como problemáticas de tipo social, técnico y jurídico, que deberemos superar para garantizar un uso eficaz del metaverso en el ámbito sanitario. En términos generales, el futuro del metaverso es prometedor en el campo de la medicina, aunque es necesario desarrollar nuevas leyes que regulen específicamente el metaverso, con el fin de superar sus posibles inconvenientes.
